# New Elements of Operative Surgery

**Published:** 1848-02

**Authors:** 


### REVIEWS.

---

ART. VI.—*New Elements of Operative Surgery.* By ALF. A. L. M. VELPEAU, Professor of Surgical Clinique of the Faculty of Medicine of Paris, Surgeon of the Hospital La Charitè, Member of the Royal Academy of Medicine, of the Institute, &c. Carefully revised, entirely remodelled, and augmented with a Treatise on Minor Surgery. Illustrated by over 200 Engravings, incorporated with the text. accompanied with an Atlas in quarto of twenty-two plates, representing the principal operative processes, surgical instruments, &c. First American, from the last Paris edition. Translated by P. S. TOWNSEND, M.D., late Physician to the Seamen's Retreat, Staten Island, New York. Augmented by the addition of several hundred pages of entirely new matter, comprising all the latest improvements and discoveries in Surgery, in America and Europe, up to the present time. Under the supervision of, and with notes and observations by, VALENTINE MOTT, M.D., Professor of the Operations of Surgery with Surgical and Pathological Anatomy, in the University of New York. Foreign Associate of the Acadèmie Royale de Mèdecine of Paris, of that of Berlin, Brussels, Athens, &c. In three volumes. Vol. I. pp. 914: 1845. Vol. II. pp. 1092: 1846. Vol. III. pp. 1162: 1847. New York: Samuel S. & William Wood.

[CONCLUDED FROM JANUARY NO.—PAGE 60.]

At the conclusion of our last notice, we were on the subject of reclamations.

It has always been our practice, in connection with the press as elsewhere, to speak precisely what we thought without reference to any claims save those of truth and justice. We have had 'an eye single' to these claims, and in defence of them we would 'cavil about the ninth part of a hair.' We flatter ourselves, too, that we have fallen into few errors during our connection with a public journal; and "we pride ourselves on this," just as Townsend and Mott pride themselves on their ability to write English. Still we are but human, and 'to err is human.' Our freedom from error hitherto, does not render the commission of one less mortifying to us, although it may serve to mitigate punishment. We have now to acknowledge an error of fact in our former paper; and we make the acknowledgment, and are going to make the atonement, with as good a grace as possible—not grudgingly like Townsend, but rather with the serene and majestic magnanimity of "Mons. V. Mott."

As we have said, when our last paper went to press we were discussing Townsend *de reclamationibus*. On what we thought sufficient grounds, we had abstracted with all possible tenderness one of the "many plumes" that decorate M. Mott's "own honored brows," and "affixed it" where we thought "it rightly belonged." We did not "*affix* it *upon* the *names*" of those to whom it rightly belonged, for three reasons: 1, because we did not know their names; 2, because it would have been difficult so to "affix it" if we had; and 3, because we were clearly of the opinion that a "name" with a "plume" sticking "upon" it or in it would not look very well. We had claimed, or rather reclaimed, for the steam-doctors and the Amphictyons of Alabama, the discovery of a "medical university" or "university in medicine." But a friend of ours (whose modesty, second only to M. Mott's, forbids our mentioning his name) curious in such matters, and solicitous for the feathers in M. Mott's cap, has carefully compared dates and satisfied us that we were in error. To "that great surgeon, Dr. Mott," then, *unqestionably belongs "priority" in this "noble discovery."* This "new path" was first "struck out" by him. "We say cheerfully with all our heart, *palmam qui meruit ferat.*"—(Townsend, *de reclamat.*, vol. ii, p. 883.) At the same time it is but just to the Steamers and the Amphictyonic council of Alabama to award to them the "merit of originality in the conception," for they certainly could not have had inkling of Mott's discovery. Whether their "process" for a "medical university" is superior to M. Mott's, we are not able to determine, inasmuch as we are unacquainted with the "details" of either. We shall possibly be able to give an "appreciation" by the time "the second edition" of this "first translation" is published.

Thus we have replaced this "plume" whence we abstracted it, and as we performed the deracination just as gingerly as possible, we are sure it will take root again, will "repullulate," if the principles "of what may be emphatically called *new surgery*" are properly applied. If John Hunter successfully transplanted a cock's spurs into a cock's comb, and if dentists successfully replace teeth they have unwittingly removed, why should not we succeed? Heaven forbid that any mistake of ours should spoil the integral beauty of Mott's plume-crowned head! On the contrary, it shall not be our fault if each one of the "plumes" on "his honored brows" does not out-dazzle that which Alexander wore at the battle of Granicus; it will go hard with us if the head-splendors of "the great surgeon of America" do not vie even with the tail-splendors of the bird of Juno!

And here it is right that we give a caution to all those who may have "reclamations" to make. They must approach the translator in "a respectful" manner; for he is a sort of male Ann Royal with another 'black-book.' They must present themselves to him with a deferential bow; for he is a surgical Warwick, making and unmaking kings at pleasure. They must stand with drooped head and veiled eyes (like the Peri at heaven's gate in Meadow's picture); for he is a new St. Peter, sitting at the celestial portal "of Practical, Operative, and Pathological and Anatomical Surgery" —a self-constituted guardian who, with his flaming sword, warns off and keeps out all those that would intrude themselves into that surgical paradise. We thank our stars for having been able to set ourselves right so soon, otherwise in the "second edition" Townsend would have burst upon us

'Fierce as ten furies, terrible as hell!'

Hear him in a sentence that bears his unmistakeable marks —one that could be sworn to in any court "in any country," Christian or Pagan!

"In conclusion on the subject of *reclamations*, and without deeming it at all necessary to pay the slightest attention to some puerile and insignificant pretensions which have been set forth and vehemently insisted upon in sundry *professed* notices and maudlin criticisms on the two first volumes, we will here barely state, that in a respectful [!] communication transmitted to us from Dr. Samuel S. Whitney, under date of Dedham, (Mass.,) March 5th, 1846, he claims the honor of having been the first person,—and before Dr. Ruschenberger of the U. S. Navy, to whose communication (see Vol. II of this work,) he alludes,—in this country at least—who clearly diagnosed a case of *unequivocal* aneurism of the *basilar artery.*"—Preface, vol. iii, p. vi.

"In conclusion of reclamations" indeed! Why the poor man has not yet seen the beginning of the end! Sancho Panza after being blanket-tossed, and Nick Bottom with his ass's head after being fairy-pinched all night, were not half so sore as will Townsend be before he can get out a new edition. Guatimozin's bed of live coals would be one of eider-down to him. He will be

> "Twixt upper, nether, and surrounding fires!"

Pharaoh, with his flies and frogs, or

> ——————'when the potent rod
> Of Amram's son, in Egypt's evil day,
> Wav'd round the coast, up call'd a pitchy cloud
> Of locusts,'—

was not so plagued as must be the translator of this unfortunate book. He need not 'begin to harden his heart' so soon; he must not 'kick against the pricks' that are now pointed at him; he must not cry 'peace, peace! when there is no peace!' He will have the consolation of knowing that all this was brought on by his own superabundant arrogance and pretension—in this wise:

In furtherance of the great end—the *motif*—of this translation—viz: the glorification of "Mons. V. Mott," all unclaimed surgical processes, floating about in the profession, are forthwith seized by Townsend, impounded like so many stray animals, and branded as Mott's—"the *onus probandi*," as Mott himself says in his letter to Liston (vol. ii), "resting with others" or with those who deny his claims. All "the great and master operations" whose originators are unknown, were "first" performed by Mott "in any country," or "in America;" all the "new discoveries and new improvements" not patented, were "created into existence" by the same "incomparable genius;" all the "new paths" not laid down in the charts, were first "struck out" (in backwoods parlance, *blazed*) by the same "great surgeon," directly or indirectly; that is to say, if he did not do all these things with his own hand, he did them by that of his pupils—on the legal principle, '*quinam facit per aliam*, *facit per se;*' and what he did not thus first do, directly or indirectly, he has done oftener and better than any body else. Take for example the cure of *naevi materni* by cauterization with red-hot needles. This is mentioned six times in the first volume and five times in the second, without including the general index of vol. iii, and "contents" of vols. i. and ii. First we have it as "Dr. Mott's process," in text, "contents," and "general index;" then it is the "American process, as performed by Dr. Mott and others," whose author (although mentioned in vol. ii by Velpeau) our translator in all his "severe researches" has never been able to discover. Then, after Dr. Post in his communication tells him that it originated with the late Dr. Bushe, of New York, he insists that Dr. Mott has performed it oftener, better, and with more success than any body else, and still persists in the table of "contents" to vol. i and ii, and in the general index in the last volume, to call it "Dr. Mott's process!" Thus he 'annexes' everything to Mott. In short, he is determined that his preceptor's "honored brows" shall have "plumes" enough, even though some of them are borrowed.

We were about to leave the prefaces, which we have perhaps *mined* sufficiently (though they are as inexhaustible as an Illinois coal-field) when we saw a new phase given to this translation, a new office ascribed to it. Already it has been presented to us as "an inexhaustible arsenal or armory," "a storehouse," "an edifice," and "a precious treasure," containing in each of these forms

'Quæ sint, quæ fuerint, quæ mox ventura trahantur.'

But we perceive that it is also "a great *organum!*" Here, now, is something very classical, very mysterious, and at the same time something very grand—as Dominie Sampson would say—'pro-di-gious!' What it is precisely, 'must give us pause;' for Townsend, like Master Slender, is afflicted with a certain ambiguity of expression, at times a little inconvenient. Yet as the Welch parson said of Master Slender, 'the faul' is in the 'ort'—'his meaning is good.' At first we thought it might mean Bacon's great work (*Novum Organon*) turned into surgery; but this is too 'far-fetched and fanciful' even for Townsend. Then, M. Mott, in his epistolary preface, evidently regards the original as a great something, whereby he is to be eternized with much ease; he speaks of entering his name as "a *compagnon de voyage*" with the great Frenchman; but whether he considers that they are going down to posterity in an omnibus, a four-horse post-coach, or a steam-car, we do not know. On the whole, this explanation is not satisfactory. Going back then to Townsend, and recollecting the pæans constantly sung by him to Mott, Velpeau, and himself, we are disposed to think he meant to signify here a *musical instrument*—the more so as this is consonant with the most frequent use of the word "*organum*" by classic authors. But this only puts the difficulty a little further off; for it is almost impossible to say whether he meant a flute, fiddle, or flageolet, cornet-a-piston or clarionet, trombone or trumpet, harp, haut-boy, or hurdy-gurdy, bassoon, bass-viol, or bass-drum. It is not easy to determine whether he meant a wind-instrument—such as that wherewith the walls of

Jericho were blown down, or a stringed-instrument such as that wherewith the walls of Thebes were built up. On the whole we incline to the belief that in his quaint way he meant to compare this translation to a *lyre*—like that which Hermes constructed out of a tortoise-shell, and gave to Apollo as a *placebo* for the loss of his cows.

Mott found Velpeau, as 'cow-stealing' Hermes found the tortoise,

> 'Moving his feet in a deliberate measure'

over the earth, and he cried out, 'a treasure!'

> 'A useful god-send are you to me now!
> *  *  *  *  *  *  *
> You must come home with me and be my guest;
> You will give joy to me, and I will do
> All that is in my power to honor you.'

Thus talked Mercury (according to Homer and Shelly) two thousand years ago; and thus almost word for word did Mott talk in his letter to Velpeau.

No sooner said than done. Townsend forthwith seized the Frenchman and, "under the supervision of Dr. Mott," murdered him outright—'bored the life and soul out' of him as Mercury did out of his tortoise; then over him stretched his "superadded new matter," as over the tortoise-shell Mercury stretched

> 'Symphonious chords of sheep-gut rhythmical.'

Thus was wrought 'this lovely instrument' upon which our 'Arcadians,'

> 'Et cantare pares, et respondere parati,'

alternately have kept up such an eternal clatter. First Townsend 'strook the chords' and

> ———'unlocked the treasure
> Of his deep song, illustrating the birth
> Of the bright Gods, and the dark desert earth;
> And how to the Immortals every one
> A portion was assigned of all that is;'

but chiefly it is Mott, that "christian and man of science," whom he has

> 'Clothed in the light of his loud melodies.

Then Mott—

> 'He piped the while, and far and wide rebounded
> The echo of his pipings; every one
> Of the Olympians sat with joy astounded!'

No wonder, for Mott fairly 'bangs Banagher'—

> 'Nec tantum Phœbo gaudet Parnassia rupes!
> Nec tantum Rhodope mirantur et Ismarus Orphea!'

Such is the best explanation that we can give of the meaning, origin, and uses of this "great organum."

Thus far with the prefaces. Turn we now to the text, and the labors of our brace of editors. In the form of suggestions, cautions, approvals, doubts, appendices, and "abrégès," the original is as full of "notes" as a sunbeam is of motes. The text is in many places buried under them. Looking at the "general index," we find it quite filled with "Townsend"—scarcely anything else to be seen—the remainder being "Mott"; so that what with the large quantity of Townsend and Mott, and the small quantity of Velpeau, one is irresistibly reminded of Falstaff's two gallons of sack to a halfpenny's worth of bread. These notes are of variable length, from a single intercalated word, to many pages; all of them from the least to the greatest having the writer's signature, though it would be easy enough to recognize them without this. It is curious to observe how these signatures vary according to the variant mood of the translator. Thus, when he wishes to seem particularly modest, when his pride apes humility, we have, by a sort of *diminuendo* movement, plain "P. S. Townsend," then "P. S. T.," then "T.;" then, *crescendo*, "Dr. Townsend," "Dr. P. S. Townsend," and "P. S. Townsend, M.D."—the last being retained for grand occasions, when he is in full feather.

The notes are as variable in subject as in length. Though "confined for the greater part of his life to the practice of medicine strictly so called," Townsend is as much at home in surgery, as if he had been born, baptized, and bred in it. Velpeau, after many years of devotion to it in large hospitals and in private practice, does not speak half so confidently as his translator. The latter is as intuitively wise as the seventh son of a seventh son. No speculation is too subtle, no theory too recondite for him; there is nothing too complex or too simple, too great or too small. His mind is at once telescopic and microscopic, presbyopic and myopic. It takes in the broadest and remotest outlines, it siezes the minutest and nearest detail. His spirit is like Napoleon's,

> 'antithetically mixed
> One moment of the mightiest, and again
> On little objects with like firmness fixed.'

He is profound in aneurisms and poultices, acute in hernia and adhesive plasters; he is voluble on myotomy and tenotomy, exsections and amputations; of peno-plasty, rhino-plasty, episco-plasty, and all sorts of plasty, he talks as familiarly as 'gentle maidens do of puppy dogs;' he is learned in artificial eyes and artificial anus; and while he is admirable on the advantages of cold-water irrigations, he is truly captivating on the virtues of brandy-and-water plain!

Then, what a discursive mind he has! Seutin's dextrine bandage suggests to him "a masked battery!" Galvano-puncture leads him to Morse's Telegraph (which he mentions more than once), and this sets him to prophecying, and also reminds him that "Perkins' tractors" were a "scientific contrivance" and an "American invention"—but whether "used by Dr. Mott and others," as the hot needles in nævi, we are not informed. Starting with Ancell's Essays in the London Lancet on Leibig's chemical doctrines, and his commendation of McIlwain's treatment of tumors, he wanders away off to the painting and sculpture and social life of Greece, and winds up by explaining to us how first the Greeks and then the Romans became the conquerors of the world. He is present at a severe surgical operation, and in the fortitude which the patient exhibits, he sees the power of religion over the human heart, the special providence of God, and the immortality of the soul! As both the latter passages possess some of his characteristics, especially the "spirit or perspicuity," and the "purity of diction" on which he "plumes" himself, we shall anticipate ourselves a little and quote them here.

"A learned and practical member of our profession, Henry Ancell, Esq., of London, in his report on the Progress of Surgery, (*Ranking's Half-Yearly Abstract of the Medical Sciences*, American edition, New York, 1845, vol. I, p. 222, et. seq.,) and who as one of the earliest illustrators of the great truths unfolded by Leibig, merits the lasting gratitude of the profession, (see his Discourses in the *London Lancet*,) thus speaks in eulogistic terms of the recent work of Mr. McIlwain on Tumors, (London, 1845,) inasmuch as the last mentioned author, evincing that he keeps progress with the advance of modern sciences, boldly, and as we think, truly, maintains that those morbid growths called *Tumors*, whatever be their classification, are to be chiefly ascribed to improper attention to the nature [and quantity he might have added. T.] of our food. On which account, Mr. McIlwain very naturally looks to the *liver* as the direct source of such morbid and abnormal productions. In other words, he ascribes the growth of tumors chiefly to sedentary habits, and the indiscriminate use of alcohol, and greasy, fatty, and saccharine food, because of their superabundance of carbon. *Ab inverso*, the cure or prevention lies in the abstinence from such indulgence and strict attention to exercise and regimen. Hence, says Mr. McIlwain, the inutility of topical applications. He instances even the cure of *cancerous tumors*, by rigid adherence to these rules, which was proved moreover by a return of pain at every transgression of regimen.

"[The faultless forms of organic development, handed down to us in the paintings and sculptures of ancient Greece, while they are speaking memorials of the supreme health which existed among that people, [*Qu. what people?*] receive a ready solution, in the philosophy which governed them

[*the faultless forms?*] in their rules of social life, especially in the cultivation of manly sports, athletic exercises, and games, and free exposure to the open air and adherence to a frugal diet. This made them [*whom?*] and the Romans, successively, so great a military nation, and the conquerors of the world. T.]"

See now into how small a nut-shell is compressed that upon which volume after volume has been written; and how, by implication merely, is solved a question upon which Gibbon wrote his immortal work. That Gibbon should have died of scrotal hernia before our translator lived! That Niebuhr and Arnold, "T.'s" contemporaries, should have died without knowing him!

The other passage occurs in a report of an operation on the jaw. This report, which was "read to and approved of and corrected by Dr. Mott," will come under our notice again.

"The incision upon the carotid was also brought together in the same manner by two or three sutures, and the patient let to remain on the table upon which he had been bolstered up, and where he had been operated upon. Being now, as was to be expected, somewhat pallid and languid, and the pulse greatly reduced in frequency and force, though there was no actual syncope, this was met by a more liberal use of warm wine and water. But he exhibited constantly the same imperturbable calm and resolution which he had during the operation; because he believed, as he said before the operator began, and when he took leave of some of his young friends the day before, upon a higher power than man. It was this serene christian faith and resignation which was the true secret of his incomparable and heroic courage. I have seen such demonstrations, but none of so high an order, on the dying couch, from the same blessed consolation, which none but those who have imparted to them this priceless boon, and through divine grace, can realize or enjoy. And I have often said, that if any thing were wanting to convince me of the power of religion on the heart, and of the constant supervision of the divine Creator over human actions, and the link between Him and the immortality of the soul, it would be these sublime moral spectacles in the hour of overwhelming tribulation and unutterable anguish, and when reliance, and hope alone in our Almighty Father, can disarm death and every mortal sorrow of their sting, and make us triumph over every worldly desire and the grave itself.

"The patient recovered perfectly in a few weeks, nearly the whole wound having healed by the first intention.

"P. S. TOWNSEND, M.D."

There! there is "purity of diction," and piety too! Some indeed might call it pious coxcombry, or piety run mad, or a fine specimen of cant written in the 'powerfully-weak' style. But there it is!—a specimen of the "new matter" wherewith four compact octavo volumes of French are swollen, expanded, magnified, augmented, and increased to three huge unwieldy, ponderous, and dropsical ones of *quasi* English. And it is "entirely new"—new in its conception, expression, and location; in short, new in every kind of sense.

Yet are we glad to meet with it. It has solved a question that has sorely puzzled us. We have pondered and pondered and pondered, and then after 'brief breathing time' we have pondered again, beating our dull brains fruitlessly to discover the true vocation of M. Mott's personal friend, pupil, and kinsman. He is no surgeon, that he himself tells us, just to show that he undertook this translation as a work of pure philanthrophy. Physician he cannot be, although he did for a time devote himself "to the investigation of febrile diseases as personally examined and investigated by himself;" for surgery has in these latter days monopolized the entire healing art. Physic is swallowed up in surgery just like 'Death is swallowed up in Victory.' 'Othello's occupation 's gone!' and if it were not, it is very evident that his heart was never in it; he practiced physic, when physic *was*, merely as a means of livelihood, or as a pastime, preferring even to be mischievous rather than idle. The true intent and meaning of the man was therefore what puzzled us. But now we have it, and we are at peace, we shall not be tormented on our death-bed. Townsend should have been a preacher—nay, he *is* a *preacher;* but he ought to be and was intended for a bishop—ay, and an arch-bishop. So strong is the tendency that here in the translation of a work on surgery his peculiar genius, the divine afflatus, breaks forth with a 'forty-parson power.' No 'melancholy Jacques' is he who merely sees

'Books in the running brooks,
Sermons in stones, and good in everything.'

He is something far better than this. He lives in a higher region.

——'His soul is like a star,
And dwells apart!'

See 'sermons in stones,' indeed! Many of us can do that, but it is given to the few, to the chosen ones, to the 'angels in disguise,' who, clothed in fleshly garments, walk through this 'vale of tears,' as through a place 'where for some sin to sorrow they were cast'—it is given to these only to see in a surgical operation the beauty and holiness of religion! the overruling providence of God! and the link that binds man's immortal spirit to the Great Father of spirits! Our translator is another mournful instance showing how much an accident may make or mar. With eloquence enough in him for a Bossuet and Massillon both together; with Jeremy Taylor's glowing piety and rare fecundity of language; with Tillotson's ease and naturalness; with Barrow's strength and force; with Bunyan's holy zeal and pious quaintness; with Dean Swift's wit and 'pure well of English undefiled'—with that in him

——'which might give
Light to a world, and make a nation live,'

he is engaged in translating, "and with notes," a book on practical surgery. The genial current of his soul was early turned awry—yet see what tremendous efforts it makes to get into the true channel! The god within him has ever had to be repressed—and behold with what a mortal agony he sometimes wrestles with it! Such has been 'inevitable destiny' in his case! Gentle reader! is it not a hard case!

But let us look a little while at the translation. The translator and his sponsor say that "they pride themselves on this." They congratulate themselves that they have achieved it "in a manner not entirely wanting either in spirit or perspicuity;" and then they proceed to make a grand flourish about "purity of diction." Indeed they flourish this on several occasions; it is one of the chief recommendations of the book; the "purity of diction" being so remarkable that the translation "may be deemed worthy, for a long time to come, to occupy a permanent *rank* in the *hands* of every person devoted to" [operative surgery,] "this now-absorbing and most clearly defined portion of the healing art." Nor is this all. They think it also necessary to laud the style of the original, as if that would enhance the value of their labor, or make the translation more deserving of encomium. So that, directly and indirectly, there are three things predicated of this translation: 1, spirit and perspicuity; 2, remarkable purity of diction; 3, indirectly and by way of absurdity, the habit-bitual felicitous style" and the "elegant diction" of the *original.*

As it regards the last predicate, it would be easy to prove the contrary without going beyond this translation itself. But if our delectable translator or his sponsor will make us a present of a good copy of the original, any time within a twelve-month, we will undertake to prove our denial from it, to the satisfaction of any French scholar. We are willing, however, to take it for the present at the translator's estimate, as it only makes his own faults less pardonable.

As it respects the first predicate, we have to say in the first place that the whole scheme of the translation is wrong —that the plan is erroneous, and the execution clumsy and unskilful. A distinguished author (Gœthe) says that "there are two maxims of translation, the one requires that a foreign author be brought to us in such a manner that we may regard him as our own; the other, on the contrary, demands that we transport ourselves over to him, and adopt his situation, his mode of speaking, his peculiarities." When we want matter alone, we select the former; when we want matter and form, we select the latter. And now in the case of the work under consideration, we put it to any man's common sense, if the former is not the proper one? What care we how Velpeau talked? What care we about his manner, his peculiarities? It is the matter, it is *what* he said, that we want; and we want this so presented that we may look upon it as our own, that we may domesticate it, that we may add it to the home-stock. We want Velpeau in the mother-tongue as he himself would write, if he were one of us. But Townsend and Mott pride themselves on having adhered "as closely and rigidly as possible to the original text of the author, *verbum verbo*, even at the risk of appearing, at times, to be speaking in an idiom somewhat Gallican."

*At times* in an idiom *somewhat* Gallican! We present these three volumes to the reader as *wholly* Gallican all the time! The plan vitiates the whole *ab ovo.*

But even with their own scheme the execution is clumsy and unskilful. They do not seem to have any confidence in their knowledge of French, even while they boast of it; for after translating the most common words and simple phrases, they add the French, in parentheses. The text is everywhere thus interlarded. To say the least, it spoils the beauty of the page, and is therefore objectionable; but it annoys the reader, and throws a doubt over the correctness of the translation. If this had been done only now and then, it might be borne with; but, in the whole three volumes, there is scarcely one single page which is not so disfigured. If it had been confined to instances, many or few, where there could be any reasonable doubt as to the signification given to the words, it might still be borne with; but in a majority of instances the signification of the words is perfectly obvious. To show how this interlardation looks, we present some specimens. We have not selected the *worst*, but have taken short sentences and paragraphs from different parts of each of the three volumes to show that what we have said is true.

"Article I.—Fanons.

"Fanons comprise three principal varieties: 1. The drap fanon; 2. The fanon properly so called; 3. The false fanon.

"§ I. The name of drap fanon, or splint-holder, (*porte-attelle*,) is given to a piece of linen somewhat longer than the fractured limb, and upon which the other parts of the dressing are first laid. Properly speaking, this is a simple alèze, or a napkin, destined to envelop the different objects that are placed about the fractured leg or thigh. In other respects, the use of the drap fanon is easy to understand. If, for example, it is the leg we are treating, we spread out a napkin, deprived of its hem; upon this napkin we place the strips of Scultetus; and upon these strips we place, opposite to the fracture, some long compresses. When these latter are placed upon the limb, and then fastened by the strips of Scultetus, we roll up the lateral splints, from right and left, in the edges of the primitive napkin, or drap fanon; we thus bring each splint, by degrees, up to the distance of an inch from the surface of the limb; the cushions are then introduced between the limb and the splints thus arranged; the cushion and the splint are also placed; and we then fasten the whole by means of circular bandages.

"It is easily conceived, that in treating a fracture of the thigh, the drap fanon must be of much greater length, and also that less length would be required for the arm or forearm.

"Meanwhile, the drap fanon is nothing more than the envelop of linen (*la toile d'enveloppe*) which we sometimes have occasion for in dressings somewhat complicated."

"In this respect, there are, in reality, some advantages in leaving the flap free, after it has been cut. It is then seen to retract (*revenir*) upon itself in the direction of its two largest diameters, to become thicker and firmer, (*se durcir*,) and more and more vascular, and to assume, in fact, some of the characters of the integuments of the cranium, or of the face. When it has become so, we may, without fear, pare (*aviver*) its edges, and fasten them, either by suture or bandages, on the part which is to become their new residence."

"As with the nose, it is the skin of the neighborhood which is to furnish the material for reparation, (*raccommodage*.)

"*First Stage*, (*Premier Temps*).—We commence by excising, shaping, (*régulariser*,) and paring (*rafraichir*) the mutilated border of the ear. We afterwards separate above, below, or at the posterior part of the concha, (*conque*,) the integuments which cover the temple, mastoid process, or subauricular fossa (*échancrure*) in the neck, a little nearer to the meatus auditorius than in a line with the abraded (*avive*) border, but in a direction parallel with this border."

"It results from this, that the new nose, feebly sustained above, sinks down (*s'abaisse*) more than is desirable, shrinks (*se resserre*) into the form of a tumor, and assumes a palish tint, which contrasts (*tranche*) with the rest of the visage. A patient thus operated upon by Delpech, and whom I saw at Paris, had a nose that was wrinkled (*ridé*) and shrivelled (*ratatiné*) like a potato, and which a lupus, moreover, finally destroyed."

"*Rhinoplasty by simple Separation* (*Décollement*) *of the Tissues.*—After having separated by dissection the teguments around the deformity, to the extent of half an inch or an inch, it is generally easy to stretch (*d'allonger*) the parts and draw them towards each other, (*d'attirer*,) so as to be enabled to bring them into contact (*les affronter*) by their previously pared edges."

"The dread which prevailed among surgeons of the last century, of wounding the inter-articular (diarthrodiaux) cartilages, exposing them to the air, and touching them with the instrument, is at the present day entirely dispelled. In place of all those precautions formerly recommended in order to avoid the articular surface, which constitutes the bottom of the stump, some modern surgeons go to the extent of advising that it should be wounded expressly. For example, M. Gensoul (*Thèse* No. 109, Paris, 1824,) is of the opinion of Richter and Bromfield, that, in scarifying it (la découpant) with the point of the knife, we have a better prospect of cicatrization by the first intention. This practice, adopted also by some surgeons of Paris, and which is attended with no inconvenience, seems, nevertheless, to be sustained upon a position which is far from being demonstrated. In fact, it is incorrect to say, with Béclard and many others, that after amputation in the *contiguity*, [*i. e.*, in the joint, vid. supra. T.] the smooth face of the cartilage does not unite with the flaps, but remains free even after the final cure, unless by some means or another, inflammation has been excited. This can only take place by exception. Whether the instrument comes in contact with it or not, it nevertheless contracts, and that speedily, firm adhesions with the tissues that cover it; and it is as useless to scrape it with a scalpel as to cauterize it, in the manner practiced in the time of Heliodorus. If the agglutination is not immediate, the cartilaginous surface, acted upon the cellular granulations which are formed upon the bone, soon detaches itself, sometimes in fragments, sometimes in large pieces, (plaques,) at other times in the form of a shell, (coque,) and soon completely exfoliates, leaving exposed a vermilion-colored wound, which afterwards cicatrizes with great facility. In the contrary case it does not perceptibly change its appearance; it only loses its polish and becomes rugose, (rugueuse;) but a molecular action soon develops itself, erodes, (miner,) and insensibly dissolves it, until it has totally disappeared. Constituting the true epiderm of the bones, and consisting of a simple anhiste (anhiste) tissue, it cannot, with the attributes that belong to it, exist any longer than while the articular movements are preserved. As soon as the living tissues rest permanently upon it, (la touchent à demeure,) the vitality of the bones, properly so called, begins to act upon it and to destroy it, by creating the cellulo-fibrous deposition, (couche,) which is the base of every sound cicatrix; unless in its actual state of cartilage, (véritable épichondre,) it becomes agglutinated to the soft tissues, by becoming, as M. Champion thinks, organized and blended with them."

"For superficial carious affections, whatever may be their extent, we confine ourselves to the use of the rasp. Having laid the diseased parts bare by means of the proper incisions, the surgeon, holding the cutting plate (plaque tranchante) of the rasp in his left hand, and embracing its handle with his right hand, proceeds to scrape and grate (grattera) the carious or necrosed surface, until he has completely removed it, and perceives red points (pointillé rouge) and a granular aspect, and no longer a yellow or livid tint, at the bottom of the rasped surface, (la plaque ruginée.)

"In its union with the body of the bone the head of the humerus forms an extremely open angle, (extrêmement ouvert,) and the fibrous capsule is inserted a little upon the inside (en deçà.) In the amputation it is necessary that the edge of the instrument should describe a circular line exactly corresponding (semblable) to the plane (plan) of the hand, if we desire to separate the fibrous tissues from it with facility."

"Operating on an old man for a sarcoma, I began with a horizontal incision, extending from the left labial commissure to the summit (sommet) of the corresponding mastoid process, and which I transformed into a T incision by means of a vertical one carried down to the great horn of the os hyoides. I had thus two triangular flaps, which I dissected, and turned back, the one in front and the other behind. After having sawed through the bone near the symphysis, I proceeded to detach from it the soft parts behind and underneath. Having raised up the angle of the jaw, and isolated the ramus, (la branche,) I cut through the neck of its condyle with the flat rowel of M. Martin, (j'en coupai le col du condyle avec la molette plane de M. Martin.)"

These quotations are from the first and second volumes. We were about to select some from the third volume, but desisted in mercy to our journal. It certainly never contained in the same space so much of bad translation, even including our own sins in that way, which though few were great. In the third volume, as in the first two, specimens like those we have quoted are as thick 'as leaves in Vallambrosa.' Now in all these instances, if the proper translation has been given there was no need of subjoining the word translated; if the proper translation has not been given, it was the duty of the writer to give it.

As to the "spirit" vaunted by the writer, it is evident that in all the sentences quoted, it is essentially French—the words, many of them, are English, but they are collocated and arranged in obedience to French models. The "perspicuity" claimed by the translator may be allowed in the restricted sense given to it by him, viz:—that the meaning of the author can be gathered from the translation. Yet among the paragraphs just quoted, we think there are some which will puzzle the reader. So, too, the meaning can be gathered from such expressions as "three years of old,"—"surgeons were deterred by it [opening the pericardium] from the fear of wounding the heart,"—"the needle that Scarpa rendered popular, slender, and eighteen inches long;" but certainly the perspicuity is not remarkable. These we might multiply almost to any extent.

With respect to the "purity of diction," we are embarrassed again by the unfortunate ambiguity of the translator. If by diction is meant style, it is sufficiently answered by what we have already said. If, as we suppose, it has reference to the use of English words and idioms in contradistinction to French, the claim is unfounded. If the constant and frequent use of French words as though they were the most common and familiar English, constitutes "purity," then is this translation, notes and all, pre-eminently entitled to be called pure. For everywhere we find *fanon*, *paillasson*, *plumasseaux*, *gâteau*, *ècusson*, *erigne*, *pelote*, *peloton*, *gros*, *boulette*, *bandelette*, *abrègè*, *bascule*, *mêche*, *tente*, *pacquet*, *debridement*, *dècollement*, and hundreds of others, used as familiarly as though they were the best established and best understood words in the vernacular. In fact, a reader unacquainted with the French tongue will have to peruse these volumes with the dictionary in his hands. Perhaps it is this which the translator meant by "an idiom *somewhat* Gallican." In our opinion there is no excuse for this. The French may have some advantages over the English in logical precision of terms, but we insist upon it that in quite all the cases to which we refer, well known and well established words of our language might have been and may be found. We certainly have enough of French incorporated into the language now—Heaven save us from this new influx.

The faults of which we have been speaking exist in still greater perfection in such of our annotator's "superadded new matter" as consists of abridgments of French pamphlets. It is, indeed, in the notes that the peculiar genius of the man breaks forth. There he is unfettered by grammatical rules whether French or English; there he spurns all control of logic, no matter from which side of St. George's channel it may come. We have already exhibited some specimens of the "American additions" to this translation, and we shall present some more in the sequel.

It is in these notes, too, in "the 1500 hundred pages of entirely new matter," that the chief design of this translation is disclosed, and its true object unfolded. That design was and is to pander to M. Mott's inordinate craving for notoriety. He cannot rest satisfied with his present position; 'admonished by his time of life," he cannot wait the slow process of years; he cannot trust even to a posthumous tribunal, but he attempts to forestall that tribunal. Hence it is that this translation and annotation was undertaken, and that in it Mott's name is never mentioned without a laudatory adjective. The incense of praise is burned under his nose all the time—the censer is never allowed to get cool. No occasion, small or great, is missed; no opportunity allowed to go "unprofited of," to use Mott's language. We have already referred to an instance of this in the matter of nævi materni, where Dr. Bushe's name, as the author of a certain process of cure, was studiously kept back, and where Dr. Mott's was just as studiously brought forward. This is a very small matter indeed; but there are some smaller matters than this. Thus, we are informed with the utmost gravity that "in the opinion of Dr. Mott sponge is a valuable and powerful *absorbent;*" that "Dr. Mott says stramonium leaves, alone or mingled with a bread-and-milk poultice, are a valuable application;" that "Dr. Mott remarks that the great benefit of dressing old ulcers with long strips of adhesive plaster (Baynton's method) is, that these strips give tone to the limb by their mechanical compression, repress exuberant granulations, and thereby promote cicatrization," &c. These are some of the "*discoveries* and *improvements* in surgical processes and principles" which the translator pledged himself to rescue from the "indistinctness into which they were necessarily passing," owing to Dr. Mott's "*repugnance* to self-laudation!"

But we will give another instance, which is a fair specimen. The translator, after informing us we know not how often in the first and second volumes that Dr. Mott was the first man to exsect the lower jaw, goes to work in the second volume, p. 882, to prove it. The section is entitled "claims of Dr. Mott as the originator of exsection of the lower jaw." After gently scolding Cusack and Huston, and growing facetious—we may say, indeed, funny—at their expense, he proceeds to detail at great length, several cases in which this operation was performed by his *magnus Apollo*, M. Mott. This is followed by the "report of a committee" of the New York Medical Society awarding priority to Dr. Mott. Then comes another case, the report of which occupies six and a half pages, and of course is rambling, tedious, and excessively minute. It had already been noticed by M. Mott, at an expense of two pages, in his preface to the first volume; and to make the matter still worse, all that is essential (especially after the previous reports) might be comprized in half a page. In the report (corrected by M. Mott) the writer contrives to inform us *twice* that Dr. Mott was the *first* to excise the lower jaw for *osteo-sarcoma*, (the claim being already whittled down)—adding, that he had then performed it fifteen times; he contrives to inform us *twice* that Mott was the *first* to propose tying the carotid artery in these cases—adding, that he had at that time tied the carotid, in these and other cases, twenty-two times; and he also informs us *twice* that the suggestion of the *curvilinear incision* in operations on the lower jaw is also due to Dr. Mott. Then comes Mott's letter to Liston, in which the former's claim to priority in exsecting the lower jaw is reiterated. And yet when these articles were placed in the second volume, (and at the time some of them were written) both Townsend and Mott knew that the "claims" of the latter were altogether unfounded, so far as the operation on the jaw is concerned! All that he can claim is the suggestion of the curvilinear incision. But it is hoped by this constant reiteration, this systematic *battologizing* to stifle truth, or to frighten it away; and to get a verdict in Mott's favor as it were by default.

With the same view we are continually regaled with expressions like these—"such men as Morgagni and Mott;" "such great practical observers and surgeons as Dr. Mott himself," (Mott seeming to be a dual noun, or 'a noun of multitude, signifying many'); and now and then refreshed with a splendid burst like the following:

"This belongs in fact to the great department of *Conservative Surgery*, which we have so much insisted upon in our notes in the text of this work, (see under Amputations,) and in which we are fully borne out by the opinions of Dr. Mott, than whom certainly no one could be listened to with greater attention, in what concerns the relinquishment or abridgement of surgical for milder remedies; for who has dealt so largely, and with such brilliant success, in the first-mentioned class of those resources of our art, as that great surgeon?"

It must be acknowledged, indeed, that the translator brought to this part of his labor, a most unbounded admiration for Dr. Mott. It was his "attachment to a long cherished personal and honored friend, preceptor, and kinsman," which suggested to him this translation, and especially this annotation.

But this is no ordinary *attachment;* it is a more than mortal passion. The translator quite forgets *himself* in it; or perhaps his love for Mott springs from his self-admiration—he sees in Mott his *possible* self, what he might have been if his intellectual waters like those of the Croton river had not been dyked and dammed and turned into artificial channels. There is a devotedness about this attachment which "naturally seems marvellous"—a fondness more than earthly, which is a celestial. It is a life-long passion, and, like Apollo's love for Hyacinth, is confirmed by long habit—'*longaque alit assuetudine flammas.*' It is the feeling of youth carried into age, having now youth's fervor, and manhood's earnestness and strength. But Apollo's love for Hyacinth was as nothing to it—

> 'To Phœbus was not Hyacinth so dear,

> Nor to himself Narcissus.'

The old myth of Damon and Pythias is not a type of it. David did not so love Absalom; nor Jacob, Benjamin. Dido did not so love Æneas. It is more like the love of Laura for Petrarch; and yet more like it seems to that of Don Adriano de Armado for Jaquenetta. Like that 'most heroical vassal' our annotator 'affects the very ground, which is base, where [Mott's] shoe, which is baser, guided by his foot, which is basest, doth tread.'

It is very certain that if our annotator brought to this undertaking unbounded admiration for his "respected associate," he likewise brought vast literary abilities. And, as we have just said, it is in the "notes" that his lordly contempt of logic and rhetoric is conspicuous. We have given numerous instances of this sort, in present and previous papers, but it may not be amiss to add a few more. See in the following how 'he draws out the thread of his verbosity finer than the staple of his argument,' to an extent indeed that might astonish his amatory prototype, the 'most gallant, illustrate, and learned gentleman,' Armado; but let no man read it who has not lungs constituted like those of a four-mile horse, or fashioned on the model of a pair of double-bellows, for he does so at his peril.

"Prof. Henderson, of Edinburgh, in his course of Clinical Lectures in that city, (Cormack's *London and Edinburgh Monthly Journal of Medicine*, May, 1843, pp. 443 to 457,) while treating with more clearness and good sense than most writers do, on the subject of Auscultation as a means of diagnosing thoracic and sub-sternal aneurism, as of the aorta and arteria innominata—and more especially after laying down many apparently plausible rules for distinguishing the pure and second sound of the heart, or that of the recoil of the blood in the large vessels upon the semilunar valves, from that of the murmur of its regurgitation into the ventricle, or of its passage through the aperture of the aneurismal sac—and also after specifying the danger of a false diagnosis from the impulsation of the heart, communicated, for example, through a hepatized portion of the lung, an empyema, (ossific formation and tumors, he could have added,) and that the law, as laid down or discovered by this professor, (as he seems to assert,) that an abnormal interval of time between the diastole of the heart and that of the remote arteries (as the radial, for example,) is a sure diagnosis of regurgitation of blood into the left ventricle, and therefore of incompetency of the aortic valves fully to perform their office, and consequently a proof that an operation for aneurisms of the *arteria innominata*, under such circumstances, must never be attempted—(all of which valuable knowledge, if true, for the surgeon, does not rigidly come within the limits of this work, but rather of pathological surgery, especially in the present imperfect condition of the doctrines of auscultation and percussion,) makes, however, an observation which may properly be inserted within the text of our author, as it has immediate practical reference more particularly, and also furnishes corroborative evidence to the truth of the new principles laid down in the improved mode of treating aneurism, as recently established in a most successful and satisfactory manner by the surgeons of Dublin."

The reports of operations in these volume are quite all specimens "of a diffuse or verbose amplification." We have already quoted the pious and pathetical peroration of one of of them, and we may make one or two more quotations from it. It should be remembered that this report especially was "read to, approved *of*, and corrected by Dr. Mott."

"About a year since his uncle (present in the room after the operation) told me Mr. Baker complained of pain in the right side of the lower jaw which soon began to swell and so continued until it reached its present magnitude—being apparently a uniform enlargement of the whole of the middle part of the base of the jaw on that side which, with the induration of the superincumbent tissues, periosteum, aponeurosis, fasciæ, and muscles, give it to the eye and feel the form of a spindle-shaped, hard, consolidated, and apparently almost boney or semi-cartilaginous tumor throughout, perfectly unyielding to pressure, and about *three inches* through in its transverse diameter, or that through its middle, and *five to six* inches in its longitudinal diameter or that in a line with the base of the jaw, tapering each way as it reaches the angle of the jaw at one end and near the symphysis of the chin at the other."

We like to be particular; and we are really at a loss to know whether Baker first had a pain in his jaw a year previous the operation, or whether the reporter first heard of it through the uncle a year previous to the operation. Then, passing over some minor matters, we would be pleased to know the antecedent to the relative *which*, the subject of the verb *give*, and the precise shape of the "tumor" after it gets through with its peculiar mode of "tapering." We are "the more particular" in these matters as the report from which we quote is a part of that "precious treasure for all future time," which the student is to "peruse and re peruse" unceasingly — to 'keep a keeping-on at it,' like the man's wife did 'at' scolding.

Dr. Mott, we are next informed, "pronounced this case to be one of the same kind as the twelve to fifteen others *for* which he had operated *upon* during the last twenty-six years"—a mistake of three years, by the way, but let it pass.

"He [the uncle] had never known him in fact to have suffered from any disease, and he had never had any affection whatever, except that some months back he had been attacked with a slight *erysipelatous inflammation* in one of his legs—I think he said in the calf, which, however, soon subsided without ending in suppuration or ulceration as one might have imagined it would have done, as a natural drain in a person like this patient evidently inclined to a rather gross and plethoric habit."

Without noticing the beautiful confusion of tenses and moods and pronouns in this little sentence, it is respectfully submitted that no man has a right to trifle with his readers as we are trifled with here. What business had the narrator to say anything about the calf of the man's leg, unless he knew that the inflammation had been seated in the calf? What right had he to *think* about it at all, when certainty was required and was within his reach? As it stands he might as well have said 'in a horn,' or 'in a spoon,' so far as information is concerned. We are glad, though, since he would say, "I think he said in the calf," that he got into a difficulty with the next word, *which*. Your relative pronoun is a precious ugly neighbor.

Next we may give from the same report a very lucid specimen of "pathological and anatomical surgery"—we know not what else to call it.

"I asked him particularly if the erysipelas had ever attacked his face and head in the form of *St. Anthony's fire* as as I could readily conceive that in the form of *angioleucite*, involving, as it does, the thick, muscular, and aponeurotic and periosteal tissues and the bones themselves at the base of the cranium, causing distressing pain and tension of those parts, this serious variety of erysipelas (so well described by M. Velpeau in vol. I of this work) might result, especially when abundant sanguinous and cathartic depletion had not been made use of, in precisely such a disease as this osteo-sarcoma."

A few pages back we took leave of the prefaces, but we cannot resist the temptation to give here, by way of variety, two more quotations from them. The first is from the preface to vol. i. The writer is speaking of his "concluding American appendix:"

"In that also we have endeavored to do full justice to our own countrymen, for all they have contributed in [Qu. *to?*] this great department of surgery; and it is with pride we say, that they have not been behind-hand in following the example of our trans-atlantic brethren of France. Germany, and England. Two communications of this nature, of a very instructive character, have been inserted in that Appendix from Drs. Watson and A. C. Post, both surgeons of the

New York hospital. We have also in that Appendix drawn copiously from the recent valuable works of MM. Serre, Malgaigne, Pancoast, Mütter, etc."

Will somebody please to tell us if Serre and Malgaigne are "our countrymen?" 'Misery makes strange bed-fellows;' and certainly, miserable writing does no less.

The other quotation is from the preface to vol. iii. The writer is speaking of medical cliniques:

Dr. March, an esteemed Professor in the Medical College of Albany, claims priority on this subject—by a year or two in advance of Dr. Mott. We are ready to concede to him all the praise he is thereby entitled to. But we will observe, that so far as public attendance and surgical operations, and medical prescriptions for the out-door poor soliciting advice at hospitals, dispensaries, etc., goes, the matter of *Cliniques* (applied in an inverse sense to the truth so far as the etiology of the word or *bed-side* practice is considered,) is quite an old affair all over the world, having been in common vogue for a century, or for half a century at least."

We are almost disposed to stop, and to waste no more paper on an author who writes *goes* for *go*, *common vogue* for vogue, and who does not know the difference between *etiology* and *etymology*. This is quite as bad as mistaking a *punk* for *spunk*, and writing Physick's name without the *k*, through two volumes.

We have presented our translator under several aspects; in all of which we are satisfied that he was admired——by himself, if by no one else; but we have now to present him as an operator. Though professedly and confessedly no surgeon, he occasionally assumes the surgeon's office, just as sometimes a man 'assumes a virtue though he has it not.' In the instance to which we allude, he operated on a bursal tumor. To his account of the operation he has given something of a dramatic turn quite peculiar to himself. We quote from the "third and last volume;" he has previously spoken of these tumors and his *peculiar* mode of operating several times—so often that he himself has become ashamed, and apologizes for mentioning them again. For us this apology was unnecessary—we supplied it. We perfectly understand the amiable disposition that our translator has to magnify the little, to exalt the mean, to raise the low, and to support the weak. Though certainly capable, as we have said, ("vid. supra,") of comprehending with equal ease 'all things both great and small,' he has a decided proneness, a proclivity, to look at small things through the same glasses which he looks through at M. Mott—and these glasses magnify enormously. But to the operation, which he calls *écrasement*, or in the mother-tongue crushing or percussion. It will be admitted, we think, that bursal tumors stand no sort of chance with him, that he is 'on' them what some doctors are 'on fits.'

"Thus have I perfectly succeeded in a large olecranal *bursa*, which had been growing for a year or more in H., a healthy mulatto (part Indian and part white) of sound constitution and good habits, and aged about 35. The patient, who was confidential porter of a distinguished mercantile firm of this city, finding the tumor at length had attained such dimensions, being oval shaped and of the size of a small hen's egg and exceedingly tense, though elastic, as to give a considerable degree of pain and annoyance in the use of his arms in hoisting and carrying boxes and bales of goods. He had imagined his arm would have to be *amputated*, and having promised, if it should be found necessary when I should examine it, (for I had not yet seen it,) that if so serious an operation as amputation should be required it should be performed, I sent word to him to call upon me, and in that event I would give him a note to an eminent surgeon, who would do it at his clinique at the University. Immediately on looking at it I perceived it was nothing more than a bursal tumor, and as there was nothing to prevent proceeding at once to the mode of cure I have mentioned, I asked him to stretch his arm out in pronation firmly upon the table, and to turn his head away. Having at this time purposely in my hand a heavy quarto volume which I appeared to be engaged in perusing, I suddenly came down upon the enlargement with it, holding it in both my hands, with all my force, from an elevation of three feet, striking such a blow upon it as dispersed in an instant every vestige of disease. To the patient it naturally seemed *marvellous;* and in fact would have appeared to be such in the eyes of most persons out of the profession. If an operation of this kind, so instantaneous, so bloodless and painless too, it may be said, (for the pain is but momentary.) and yet so radical in its total extirpation of the disease, was known to the school of Esculapius, we cannot wonder why the ignorant, marvel-loving, superstitious multitude, before whom this master spirit could have turned such skill to a valuable account, (by momentarily taking the patient for a few instants out of their presence,) should have deemed him more than mortal, and built altars and temples to his honor.

"Far be it from the writer of this, however, to glorify himself on such an achievement, so long as its common utility and the facility with which any person may perform it, are so obviously sustained on the plain principles of common sense."

Of course not—he, the most modest of translators, annotators, and reporters! Yet if 'we who have free souls' should "momentarily" put the crown on him "for a few instants," let him not, like Cæsar on the Lupercal, thrust it aside. The second sentence, like its author, and his townsman, the great Andrew Jackson Allen, 'is itself alone.' The third sentence is a fine specimen of the writer's "perspicuity" in its enlarged sense, viz: purity, propriety, and precision. The fourth gives us "T." preparing for action; the fifth presents him in full action: both are worthy the profoundest study of the young surgeon and the young artist. The uncertainty whether the operator grasped the book with all his force, or brought it down with all his force on the tumor, will give room for the exercise of ingenuity. Instead of a heavy quarto, which may not always be at hand, it is suggested that one of the three volumes of this translation may be used, as they *will* always be at hand. Though not quartos, they are—to use one of the translator's expressions—the "ugliest kind" of octavos, and will prove quite *heavy* enough. In the seventh sentence we cannot determine whether the writer wishes to say how Esculapius did humbug, or how he might have humbugged, the people of his day. Taking the former meaning, a question suggests itself: whether it be possible that by metempsychosis the soul of Esculapius now inhabits the body of P. S. Townsend? It is an awful question, after all that we have said!

So much for the translator. '*Superat pars altera curæ*' —viz: "Mons. V. Mott," that eighth wonder of the world,

> 'Quel grande alla cui fama è angusto il mondo,
> Per cui Townsend ebbe in terra amor celeste.'

We need not occupy much time with him here. He is to be held responsible for all the offences perpetrated in this book, including the high-wrought panegyrics on himself (his repugnance to *self*-laudation being extreme) on the same principle that Townsend gives him credit for certain operations and processes—'*quinam facit*,' etc. The translation was suggested by him, it was executed under his eye, and partly by his aid, and moreover it inures to his benefit. Whatever faults (*if any*) the work has, belong to him—they are his by adoption—they "meet with his entire sanction and approval." If he should object to such a pack of sins as Townsend has fastened on him—a pack far greater and more burdensome than that of Bunyan's pilgrim—he will at least not object to carry his own pack, he will not attempt to shuffle it off his shoulders.

In our first paper, we presented some extracts from Dr. Mott's *epistolary* preface which was one of the chief attractions of the first volume, and the only production of his pen contained in that volume. We go now to the second volume, wherein we find "the detached fragments of the scaffolding of that extended work" abandoned by M. Mott. We present first some fragments from his "own special chapter on Aneurism," marking with italics that to which we desire to call attention. The writer is speaking of the *primitive carotid:*

"We were present at Sir Astley Cooper's second attempt to tie this artery, and the issue was fortunate. Dr. Wright Post was *also* the first who succeeded in this operation in this country. I have tied the primitive carotid for aneurism, and for various other purposes, TWENTY-THREE times, *most of which*, all in fact, but two, have terminated favorably. Two of these *cases* were *for* nævi materni, i. e., aneurism by anastomosis, one an infant of three, and the other of six months, and in both a radical cure of the disease was affected."

That is one fragment of this "precious treasure for all future time." We could not blame a "student," however, that should object "to peruse and *re*-peruse" such an awkward sentence. Here is another fragment:

"There is the best ground for hope that the *ligature* on the common carotid upon the cardiac principle, will continue to be a very successful *operation*, in consequence of *its* giving off no branch whatever throughout the whole length of *its* trunk, a most curious, interesting and important fact."

Very curious, indeed; no matter whether it refers to the "ligature," the "common carotid," or the "operation." M. Mott sometimes gets indignant, and then he vents his indignation after this manner:

"The extravagant, depletory, and starving *system* of Valsalva, in aneurisms, and of other practitioners for other diseases, *deserve* to fall, as *they* are doing rapidly, into disrepute."

"It is one thing," says M. Mott, "to describe, and quite a different thing to perform, an operation"—which oracular saying may be thus exemplified:

"The brachial or humeral, from its superficial course, first along the inner edge of the coraco-brachialis, and then the inner edge of the biceps, *makes a ligature* upon it an easy *operation* to a very ordinary surgeon."

Let us go now to the third volume, and see if M. Mott, since the first two volumes were published, has improved any in his felicitous manner of expressing himself. We find seven pages of concluding remarks gracefully winding up "this first American edition," and to these seven pages our attention is especially directed in the preface. The improvement hoped for, will not be found.

"I regret that the knowledge of myotomy we now possess was not at that time understood, for I am persuaded, that if I had then known as much of the benefit of dividing or excising muscles and tendons, *which we now have*, I might have restored this interesting young lady."

But enough—we fear that the reader who has accompanied us through two papers, and has not forsaken us this far in the third, is wearied. We could go on for many pages—we had almost said forever, and then not exhaust these volumes; but sooth to say we grow tired ourselves, and, what is far worse, have become so infected by bad examples that we are in danger of falling, and perhaps have fallen, into some of the very errors that we have been reprobating. It has given us no pleasure to do what we have done, or to say what we have said; especially so far as M. Mott is concerned. On the contrary, we have felt humiliated. It so happens that his name is the first American surgeon's name that we ever saw and recognized as such in an European work. This was a good while ago, before we touched even the threshold of medicine; but we recollect how leaped our heart at seeing the name, with what a glow of pride we looked at it. It was not then *M.* Mott, nor *Mons.* V. Mott; it had no French frippery or trappings of any sort about it;—*M.* Mott had not then *traveled*, had not yet learned to 'lisp and wear strange suits,' had not yet become sophisticated;—but it was plain *Mott*, like as the name of one long and well known in surgery, like as the name of one throned in the hearts of surgeons. Is it not then humiliating to see one whom we learned to regard as so justly distinguished, stoop from 'his pride of place' so low as he has here stooped? We look upon these volumes as the veriest piece of literary and professional quackery that has ever issued from the American press. This is strong language and it may be bold language, but it is also true.

Does the reader yet know, after all that we have said, what he will find in these volumes? We will tell him; but first we will tell him what he will not find. He will *not* find in the translation, from the title-page of vol. i. to "finis" of vol. iii. inclusive, one single page—certainly not three consecutive pages—of good English; he will *not* find in the "notes," in the "American additions," three consecutive *sentences* of *decent* English—and we doubt if either of the annotators can write three such consecutive sentences. But he *will* find the worst translation that *we* have ever seen; he will find worse offences than any we have pointed out against all canons of good taste, against all rules of grammar, all principles of logic, all precepts of rhetoric; above all he will find everything that Mott has ever done, and some things he did not do, detailed at full length, and repeated again and again, and inserted in all possible places and in some where it would not seem possible; he will find the celebrated Mott claiming for himself "priority and originality" for certain processes and the modification of processes, just as if he were claiming priority and originality for some modification of a patent washing-machine, a corn-sheller or a sausage-stuffer; he will find, as we have found, a good book prostituted to the advertising of what Mott has done in order to show what Mott may do! And now that one, who has had the boldness to undertake what Mott has undertaken, and the skill to accomplish what he has accomplished, should even wink at, much less aid, abet, encourage, and sanction what we have named—is it not monstrous! But we are repeatedly told that M. Mott felt that all this was due as much to his country as to himself. Wonderful man that has such a country to interest itself in him! Wonderful country that has such a man in whom to interest itself!

'O fortunatam *nimium*, sua si bona *nôrit*!'

It is confidently asserted that no practitioner or student can possibly do without this work. The practitioner will find it useful; and so will the student, if by student is meant one who has finished the curriculum of studies in medicine and surgery. But this concession applies only to the book as it came from the hands of Velpeau. The translator and his "respected associate" have done all in their power to destroy its *original* usefulness. No man, who has not the book before him, can conceive of the immense mass of crude, undigested, irrelevant "notes" that they have added. They seem to have opened all the pigeon-holes about their office, and scattered the entire contents, helter skelter, through these volumes, trusting that by some sort of affinity like would cleave to like. The edition from which they translated was published in 1839. Does any man in his senses, or one indeed a *little* out of his senses, believe that it required, or would require, fifteen hundred pages to state every thing of value that has occurred in surgery during the last seven years, even including the "discoveries" and "new improvements and views in surgical processes and principles" of "Mons. V. Mott?" Five hundred pages—nay, three hundred—would be amply sufficient. To make out the fifteen hundred pages, we have case after case copied entire, heading and all, from medical journals; we have abridgments, or, in the *lingo* of the two annotators, "abrégés," of long-winded discussions at the French Academy; we have copious quotations from the journals of the day; and we have reports of cases, with that most useless of all things, a detail of symptoms as they appeared day after day, for weeks, at morning, noon, and evening visits. "A *systematized* treatise" on the elements of operative surgery, is, to be sure, a beautiful place for all this! Had Velpeau no misgivings when he demurred to the inquiry as to whether he intended publishing a new edition? It was necessary, however, to do something to make the book correspond with the *solecism* of the title-page touching "*entirely new* matter." Does the republication of cases reported by or for M. Mott twenty and thirty years ago (all that possessed any importance having been noticed in the text by Velpeau) come under the head of entirely new matter? What if other American and all the English surgeons were to do the same thing? Our annotators do not know that a 'great book is a great evil.' They seem to think that the value of a book increases with the size and weight. They are like the foolish bird, in the Spanish fable, which thought the pumpkin a better fruit than the grape, because forsooth it was larger! We commend the moral of the fable to them—to Townsend especially, as he may have read it somewhere "in the West Indies:"

> 'Grande es, si es buena, una obra;
> Si es mala toda ella sobra.'

